# 

**Published:** 1885-02

**Authors:** 


### BOOKS AND PAMPHLETS RECEIVED.

*The International Encyclopedia of Surgery.* A Systematic Treatise on the Theory and Practice of Surgery by authors of various nations. Edited by John Ashhurst, Jr., M. D., Professor of Clinical Surgery in the University of Pennsylvania. Illustrated with chromo-lithographs and wood cuts; in six volumes. Vol. V., New York: Wm. Wood & Co.

*The Story of My Life.* By J. Marion Sims, M. D., LL. D. Edited by H. Marion Sims, M. D.; 1884. D Appleton & Co., New York.

*Biennial Report of the Alabama Insane Hospital*, at Tuskaloosa, Ala., for the years ending 30th September, 1883 and 1884.

*The Dry Treatment of Chronic Suppurative Inflammation of the Middle Ear: Muriate of Cocaine in Ophthalmic Surgery*; both by C. J. Lundy, A. M., M. D., Professor of Diseases of the Eye, Ear and Throat in the Michigan College of Medicine, Detroit. Reprints from various journals.

*A Contribution to the Relations of Ovulation and Menstruation.* By A. Reeves Jackson, A. M., M. D., Professor of Gynecology in the College of Physicians and Surgeons of Chicago. Reprint from Journal American Medical Association.

*On Oxygen as a Remedial Agent.* By Samuel S. Wallian, A. M., M. D., member of the Essex and Franklin County Medical Societies; member of the Medical Association of Northern New York; corresponding member of the State Historical Society of Wisconsin; delegate to the American Medical Association, etc.

*Transactions of the Medical Society of the State of Pennsylvania*, at its thirty-fifth annual session. Held at Philadelphia May 14, 15, 16, 1884; volume XVI; published by the Society. John G. Lee, M. D., Corresponding Secretary, Philadelphia.

*Notes on the Opium Habit.* By Asa P. Meylert, M. D., member of the Medical Society of the County of New York, etc. Third edition. Revised and enlarged. New York and London. G. P. Putnam's Sons; 1885.

*A Text-Book of Hygiene.* A comprehensive treatise on the principles and practice of preventive medicine from an American standpoint. By Geo. H. Rohe, M. D, Professor of Hygiene, College of Physicians and Surgeons, Baltimore; member of the American Public Health Association; of the American Dermatological Association; of the Medical and Chirurgical Faculty of Maryland; corresponding member of the New Orleans Academy of Sciences, etc. Baltimore: Thomas & Evans, 1885.

*The Basic Pathology and Specific Treatment of Diphtheria, Typhoid, Zymotic, Septic, Scorbutic and Putrescent Diseases Generally.* By Geo. J. Zeigler, M. D., late Physician to the Philadelphia Hospital; member of the Philadelphia County Medical Society; of the American Medical Association, etc; author of "Zoo-Adynamia," "Researches on Nitrous Oxide," "Natural Laws of Marriage," etc. Philadelphia. Geo. J. Zeigler, M. D., 1884.

*The Role of Bacteria in Infectious Diseases.* By Henry O. Marcy, A. M., M. D. Boston, Mass; President Boston Gynecological Society; late President of the American Academy of Medicine; member British Medical Association, etc. Reprints from Journal American Medical Association.

*School Hygiene in Relation to its Influence upon the Vision of Children; or, School Sanitation.* An address delivered before the Medical Association of Georgia; 1884. By A. W. Calhoun, M. D., President.

*Sixteenth Annual Report of the Presbyterian Hospital,* in the city of New York, for the year ending September 30, 1884. Compliments Hunter P. Cooper, M. D., Assistant House Physician.

*Physicians' Daily Pocket Record,* comprising a visiting list, many useful memoranda, tables, etc. By S. W. Butler, M. D. Nineteenth year. New and thoroughly revised. Edited by D. G. Brinton, M. D.; Philadelphia. D. G. Brinton, M. D., 115 S. Seventh street, 1885.

*Modern Medical Therapeutics,* a compendium of recent formulæ and specific therapeutical directions, from the practice of eminent contemporary physicians, American and Foreign. By Geo. H. Napheys, A. M., M. D. Edited by Joseph F. Edwards, M. D., and D. G. Brinton, M. D. Eighth edition, revised and enlarged. Philadelphia; D. G. Brinton, 115 S. Seventh street, 1885.

### OUR ADVERTISERS.

*Dr. J. W. Lowell & Co., Portland, Maine.*—See advertisement of this firm, and write for handbook containing information of caulocorea.

*Magnus & Hightower, Atlanta.*—Are prepared to fill orders for hydrochlorate of cocaine, the new anæsthetic, and for fresh vaccine matter.

*Drs. Taliaferro & Noble, Atlanta.*—We commend with pleasure the private infirmary, for the treatment of diseases of women, presided over by these gentlemen. It is well arranged and handsomely furnished throughout with every convenience that could be desired for the comfort of the patients.

*Surgical Instruments.*—Isaac Phillips, the instrument dealer on Broad Street, has, in addition to his large stock of instruments of other manufacturers, a full line of Tieman & Co.'s celebrated surgical instruments. Physicians can always find what they want at this house, at manufacturers' prices.

*Rio Chemical Company, St. Louis.*—Among the new advertisements in this issue of THE JOURNAL will be found one of this Company. Their preparation of "pinus canadensis" has the indorsement of the most prominent gynecologists in the land. Prof. Taliaferro, of this city, is using it in his clinics, and expresses himself as being highly satisfied with results obtained.

*Reed & Carnrick, New York.* By reference to our advertising pages it will be seen that these gentlemen are sole manufacturers of peptonized cod-liver oil and milk, and beef peptoids. This latter preparation we have used frequently, and unhesitatingly recommend it as a valuable nutritive food for invalids and convalescents. We doubt not the peptonized cod-liver oil and milk will prove a valuable preparation of this much used remedy.

*Change of Schedule.*—East Tennessee, Virginia and Georgia Railroad, commencing Jan. 18th, trains on Georgia division will be run as follows: Leave Atlanta for Rome, Dalton and Chattanooga 12:55 noon; leave Atlanta for Rome, Dalton and Chattanooga 11:10 p. m.; leave Atlanta for Macon and Florida 3:25 p. m.; leave Atlanta for Macon and Florida 4:45 a. m.; arrive Atlanta from Chattanooga and Rome 4:35 a. m; arrive Atlanta from Chattanooga and Rome 3:00 p. m.; arrive Atlanta from Macon and Florida 12:10 noon; arrive Atlanta from Macon and Florida 10:55 p. m.

703

### INDEX.

A

A bad precedent 60
Abortion: an interesting case 12, criminal 360, and Infanticide in Atlanta 425
Absorbent and antiseptic cotton 407
A death from Morphine 701
A hairpin in the Uterine Cavity 637
Albuminuria, Nitro glycerine, and chloride gold and sodium in the treatment of. 56
Alumni Association Jefferson Medical College 364
A Medical College sued 319
American Journal of Ophthalmology 364
A medical fable 639
Amputation of the thigh 528
A monster 377
Anæsthesia: Extreme for obstetric operation 1
Anæsthetics, relative merits of 96
An Inebriate Asylum 556
An anomalous tumor 577
Aneurism, traumatic, ligation of common carotid, 64
Antisepsis in Ovariotomy and Battey's Operation 62
Anuria, a case of, 375
A strange case 125
Athletic sports 634
Atlanta Medical College 53
Atlanta Medical and Surgical Union 363
A verdict of manslaughter 372
A Voice from Texas 429
A wink as good as a nod 387

B

Back ache 379
Battey's Operation, a case of, 321
Battle of the Doc's, 564
Bismuth, what of 5
Blood letting, something new in 4
Books and Pamphlets received 188, 189, 302, 303, 357, 358, 363, 419, 420, 495, 548, 549, 550, 690, 691, 692
Book Reviews: Agnew's Surgery 354, A Manual of Bandaging 631, A Manual of Diseases of the Throat and Nose 493, A Manual of Obstetrics 627, A Theoretical and Practical treatise on Hemorrhoidal Diseases 630. A Practical treatise on Massage 629, A text book of practical medicine 611, Auscultation Percussion and Urinalysis 356, Bartholow's Materia Medica 355, Basic pathology and specific treatment of diphtheria 686, Contagious and Infectious Diseases 296, Drugs and Medicines of North America 302. Elementary Principles of Electro-Therapeutics 339, Encyclopædia of Practical Quotations 494, Encyclopædia of Surgery International 688, European Notes 540, Family Atlas 301, Gunshot Wounds of small Intestines 361, Hand Book of Eclampia 300, Health Hints for Travelers 356, Helen Avon 360, Henk's Atlas of Surgical Anatomy 417, Hooper's Physicians Vade Mecum 460, Index Catalogue Surgeon-General office 509, Lock Jaw of Infants, 545, Malaria and Malarial Diseases 417, Medical Diagnosis 301, Medical Record Visiting List 494, Medical Rhymes, 546, National Dispensatory 492, Practical Manual of Obstetrics 361, Sexual Neurasthenia 357, South-Side Views 357, Shakespeare as a Physician 356, Story of my life 688, Surgical Delusions and Follies 547, Transactions Louisiana Medical Society 543, Text Book of Pathological Anatomy and Pathogenesis 539, Text-book on hygiene 687, Transactions Medical Association of Georgia 622, of Pennsylvania 674, Wood's Library for 1884, 299

C

Carroll County Medical Society, action of 561

Catarrh: Chronic Nasal 273, Treatment of 278
Cases from Practice 336
Cancer of the Stomach, Diagnosis of 417
Cellulitis of the Neck 569,: Pathology of 570, Treatment of 570
Chapped hands and frosted feet, Treatment of 63
Chemistry and Therapeutics 449
Children, their management in Disease 222
Chloral, death from 295
Cholecystotomy, Experimental 336, 385
Cholera 363, Death from 433
Cerebral Congestion complicated with Chorea, from fright 521
College Physicians and Surgeons, New York 560
Cocaine Hydro-Chlorate: The New Anæsthic 513, Clinical observations upon 608, Discovery of 561, 565, Deterioration of 567,672 Report of cases operated on under its influence 586, The price of 561
Confederate Surgeons 348, 396
Conversation in office of Lady Practitioner 560
Correspondence: Hopkins *vs.* Hammond 413 484, 534, Ill shaped noses and other irregularities of the human body 537, The new Journal, its contents 230, Our New York letter, Asiatic cholera, Orthopœdic Society, Charges against Dr. Barker 668
Cows' milk as a vehicle of disease 572
Croup, membranous 656

D

Death rate in Atlanta 427
Diagnosis, Holmes on 63
Diagnostic value of the soft palate as compared with the tongue, 641
Didn't catch his meaning 434,
Diet in consumption and other morbid states 626
Diseases of children 462
Diphtheria contracted from a patient by a physician 211
Dislocation: Treatment of, of the head of the humerus 102, of the hip joint, Rules for reducing 157, Chronic, reduction of, 665
Distribution of scholarships 45
Doctors must not tell 405
Do mental shocks to the mother leave impressions on the fœtus in utero 588
Dropsy, complicated with aneurism 531

E

Ear ache, Treatment of 126
Episcleritis with degeneration of the Iris 148
Epistaxis, how to check 638
Erysipelas, Facial 191
Ether: Death from inhalation 172, In Obstetrics, notes on the use of 31

F

Fecundity, Remarkable case 19
Fever: Hemorrhagic Malarial 120, Notes on 354, Scarlet transmitted by letter 64, Typhoid 393, Wet pack in 64, Yellow fever, the micrococcus of 383
Fœtation tubal 163
Four children at one birth 460
Fractures: Immediate treatment of by plaster of paris dressing 61, Remarkable case of 82.

G

Gaston's Operation 566

Gonorrhœa, remedies in 90

H

Hernia: Strangulated 15, Umbilical 86
Hiccough, Remedy for 225,
Hot water in the treatment of nervous diseases, Therapeutical effects of 554,
Hour-glass contraction a case of, 657
Hydrophobia, Case of 432

I

Importance of frequent post-mortem examinations 468
Inebriety and Insanity, punishment a factor of 150
Infant feeding and summer complaints 110
Infanticide in Atlanta 229, 374
Infectious Diseases 437
International Medical Congress 1887, officers of 637
Irido-cyclitis 84

K

Kairin, one of the results of administration of 579

L

Leprosy, as seen in Brazil 368
Lithotomy, A summary of 99 cases 61, Supra pubic 575

M

Malaria Hæmaturia 400
Medical Association of Alabama 119, American 61, 185, 316, 234, of Florida 186, of Georgia 118, 187, 164, 695
Medical Legislation 633, in Congress 63
Menstruation, vicarious 63
Mercury 129
Meteorological Summary 562, 639, 701

Monstrosity, A child with a dog's head 126

N

Nævus, with report of cases 321
Needles. result of swallowing of 378
Nephritis, Chronic Interstitial 596
New and old code of Ethics 46
New York Academy of Medicine 489
No cure, no pay 638
North Carolina Medical Journal 563

O

Obituaries: Dr. L. P. Yandell 119, Dr. Samuel D. Gross 249, Mrs. Wm. F. Holt 314, Mr. J. T. Harrison 314, Dr. T. L. Raines 365, 381, Dr. W. C. Norwood 365, Dr. J. J. Woodward 381, Dr. E. Jaeger Von Jaxthall 381, Dr. W. G. Drake 428, Dr. L. A. Dugas 504, Dr. S. M. Bemiss 561, Dr. T. W. Gordon 635, Dr. J. G. Thomas 636, Dr. J. Marion Sims 50, Dr. T. M. Darnall 699, Dr. James Knapp 699, Dr. Henry Gibons, Sr., 699, Dr. J. C. Faget 700
Obstruction of the gall duct, operation for the relief of, 696
Our Journal 42, 317
Our Portrait Gallery: Dr. A. W. Calhoun 189, Dr. J. S. Todd 253, Dr. V. H. Taliaferro 311, Dr. H. F. Campbell 369, Dr. Eugene Foster 422, Dr. Rob't Battey 496, Dr. W. F. Westmoreland 551
Our title page 48
Ovariotomy 226, 408

P

Paniritium 35
Penis, Gangrene of 382, Double Meatus of 527
Pelvic Abcess 597
Personal: Drs. H. F. Campbell 252, 561, H. V. M. Miller 363, J. S. Todd 363, G. H. Noble 363, R. O. Cotter 363, Rob't Battey 363. Hon. J. E. Brown 364, M. Pasteur 376, Dr. J. P. Logan 349, Dr. J. T. Johnson 382, 557, Dr. G. W. Holmes 382, L. H. Peeples 636, Koch 382, Dr. S. H. Gray 430, Dr. J. F. Alexander 430, Dr. J. McF. Gaston 47, 363, 382 Drs. Taliaferro & Noble 59, 316, Dr. J. H. Logan 430, J. C. Avary 431, Dr. Thad A. Reamy 431, Capt J. C. McMichael, 561, Hon. D. A. Speer 561, Dr. Wm. Brodie 562, Dr. A. W. Calhoun 635, 699, Dr. J. B. S. Holmes 636, Mr. F. A. Scribner 637, Dr. T. O. Powell 637, Dr. W. D. Bizzell 559, Dr. R. H. Taylor 699, Dr. K. P. Moore 699, Dr. J. M. Crawford 699, Dr. E. C. Goodrich 699, Dr. B. R. Dostor 699
Pessary, removal of 81
Physiological and Pathological experiments 359
Pleuro Pneumonia of cattle 560
Phthisis Pulmonalis 193, 603
Pill, how to take 149
Press excursion 376
Professional bias in theory and practice 364
Public health 257

Q

Quinine: Danger from large doses 62, Danger of large doses 38, New method of administering to children 127, Poisoning by, in typhoid fever 331.

R

Rectal Etherization 254

S

School Discipline 225
Selling his practice 295
Septicæmia 480
Sims Memorial Fund 185
Society Reports: Atlanta Medical and Surgical Union 406, 480, 527 657, Macon Medical Society 400, 461, 521, 588, 651
Soothing Syrup, a death from 30
Southern physicians in New York 489
State Board of health 304
State Medicine 113
Stricture: Division of without subsequent sounding 77, Attempts to remove by the "roots" 434
Surgery: Antiseptic 60, Case in 269, 330, Two remarkable cases of 212
Symblepheron 391
Syphilis as a sociological problem 626

T

Tait, Lawson, Address 421
Tobacco poisoning 648
Tompson, cotton candle wick 127
Test s in perineo 147
Testimonial of a patent medicine 432
The boy and the Bone Setter 640
The Dry treatment of Suppurative Inflammation of the middle ear 17
The last fashion with Medical Journalists 368
The object of the Medical Profession, An address 65
The muscular retractive power of the bowels in strangulated hernia 344
There's money in it 637
The retractive power in the bowels in medical practice 520
Theory on the action of hot water 409
Tonsilitis 158
Too much medicine 345
Torticollis, congenital 516

Tooth ache, a remedy for 192
Transfixion of the heart, Recovery 383
Trismus nascentium 206

V

Vaccination as a preventive of hydrophobia and yellow fever 380
Veratrum Viride, Value of as a prophylactic in 574
Verdict of a Coroner's jury 693

W

Warburg's Tincture 640
Water, diseases produced by drinking 337
Wounds: Most efficient treatment of scalp 143, Post mortem prognosis in cases of 128, of the intestines 283

Z

Zymotic Agents: Modus operandi 20

### OUR ADVERTISERS.

---

We present to-day a number of new advertisements to which we invite the attention of our readers:

*Magnus & Hightower.*—These gentlemen have an advertisement in this number of THE JOURNAL which we hope will be read by every physician receiving THE JOURNAL. Physicians ordering goods from them can rely on getting what they order as cheaply as if they were present. We know whereof we speak when we say they will sell you drugs, chemical or instruments, as low as any house in New York or Philadelphia.

*Er. Lawshe.*—Attention is directed to the card of this gentleman. He has been selling spectacles for over thirty years, and thoroughly understands his business. He is sole agent for the celebrated Arundel Spectacles, manufactured at Reading, Pa. If you are in need of glasses, write him.

*Franklin Mills Co., Chicago.*—These celebrated mills are now making the best all wheat flour known to the trade. Write them for their illustrated pamphlet which shows the structure and chemical properties of wheat. It will be sent free to all applicants.

*Wm. Snowden, Philadelphia.*—This old surgical instrument manufacturing and importing house is now making a preparation of silk known as the "Iron-dyed Silk," which is fast superseding every other kind of silk for ligatures. It is now being used by leading surgeons in all parts of the country. Write to him for sample copy of the silk, mentioning THE JOURNAL, and it will be sent you free.

A number of advertisers cannot be mentioned this month for want of space.

